# The relationship between LH and thyroid volume in patients with PCOS

**DOI:** 10.1186/1757-2215-5-43

**Published:** 2012-12-11

**Authors:** Evrim Cakir, Mustafa Sahin, Oya Topaloglu, Nujen Bozkurt Colak, Basak Karbek, Askin Gungunes, Muyesser Sayki Arslan, Ilknur Ozturk Unsal, Esra Tutal, Bekir Ucan, Tuncay Delibasi

**Affiliations:** 1 Department of Endocrinology and Metabolic Diseases, Diskapi Training and Research Hospital, Ankara, Turkey; 2 Department of Endocrinology and Metabolic Diseases, Ankara University Hospital, Ankara, Turkey; 3 Demetevler 1.cadde, 81-10, TR-06200, Ankara, Turkey

**Keywords:** Polycystic ovary syndrome, Insulin resistance, Luteinizing hormone, Estradiol, Thyroid volume

## Abstract

**Background:**

Thyroid volume (TV) has been found to be associated with age, anthropometry, smoking, iodine status and hyperinsulinemia. Hyperinsulinemia is frequent finding in patients with PCOS and has associations with TV. However, the TV has been evaluated only a few studies in patients with PCOS. Therefore, the aim of this study was to evaluate the biochemical and hormonal variables in patients with PCOS comparing with the controls and their relationships between TV.

**Methods:**

This was a case–control study conducted in a training and research hospital. The study population consisted of 47 reproductive-age PCOS women and 30 control subjects. We evaluated anthropometric, biochemical and hormonal parameters as well as thyroid volume in PCOS patients and controls. Insulin resistance was calculated using the homeostasis model assessment insulin resistance index (HOMA-IR).

**Results:**

Mean age, BMI, thyroid stimulant hormone (TSH) levels and TV were similar between groups (p<0.05). The HOMA-IR and free T4 levels were higher in patients with PCOS. However, hyperinsulinemia and insulin resistance were not found to be associated with TV. Thyroid volume was positively correlated with the LH and anti TPO levels. The participants were divided into 2 groups according to HOMA-IR levels. The mean TV measurement was higher in group with higher HOMA-IR levels, but the difference was not significant in young age PCOS patients.

**Conclusion:**

In early age PCOS patients it was observed that insulin resistance had no effect on TV. In this case, anti TPO and LH have dominant effect on TV. Chronic stimulation with LH and insulin may lead to increase in TV in later stages of the PCOS diseases.

## Introduction

Polycystic ovary syndrome (PCOS) is one of the most common endocrine disorder affecting at least 5 to 10% of women of reproductive age [1]. PCOS is characterized by hyperandrogenism, menstrual disturbance, anovulation, infertility and obesity [2] and is reported to be associated with an increased risk of cardiovascular disease (CVD) [3] and early atherosclerosis [4]. Hyperinsulinemia and insulin resistance are frequent findings in patients with PCOS and have associations with thyroid volume and nodular thyroid diseases [5].

In recent studies, thyroid volume (TV) has been found to be associated with age, anthropometry, smoking, iodine status and hyperinsulinemia [5-8]. Additionally, growth hormone and growth factors such as insulin like growth factor, epidermal growth factor have been shown to affect TV [9,10]. Furthermore, endogen TSH stimulation has been shown to increase thyroid gland size [11].

Luteinizing hormone is a glycoprotein hormone like TSH and has similar alpha subunit. Additionally, the LH has been identified to increase thyroid adenylate cylase activity [12,13]. It has been known that the LH levels are higher in patients with PCOS compared with the controls even in follicular phase [14,15].

The goiter prevalence is more common in women than in men regardless of population [16]. The higher incidence of thyroid diseases in women is previously attributed to the higher estradiol levels. Estradiol has been shown to enhance proliferative and mitogenic activities of thyroid cells [17,18]. But, in recent years chronic estradiol treatment has been shown to reduce volume densities of follicles, follicular epithelium and thyroid gland [19,20].

Therefore, the aim of this study was to evaluate the biochemical and hormonal variables in patients with PCOS and their relationships between TV.

## Materials and methods

We studied 47 patients with PCOS and 30 age- and body mass index (BMI) - matched healthy controls. We studied in a mild iodine deficiency area. The protocol was approved by the local ethics committee. All patients gave a written consent. All patients were female and non-smokers. This was a prospective case–control study conducted in a training and research hospital between September 2011-March 2012.

The diagnosis of PCOS was made according to the Rotterdam European Society for Human reproduction and Embryology/American Society for Reproductive Medicine–sponsored PCOS Consensus Workshop Group [21]. The revised diagnostic criteria of PCOS are as follows, with at least two of the following being required;

1. Oligo and/or anovulation that is menstrual disturbance

2. Clinical and/or biochemical signs of hyperandrogenism

3. Polycystic ovarian appearance on ultrasound

The control group (n=30) consisted of healthy patients who were admitted to check-up unit without any systemic disorder. All of the women in the control group had hirsutism score <8. All women in the control group had regular menses, every 21–35 days. None of the women in the control group had polycystic ovary on ultrasound.

Those participants who had a smoking history, diabetes mellitus, hyperprolactinemia, congenital adrenal hyperplasia, androgen-secreting tumours, thyroid disorders including thyroid parenchyma heterogeneity and/or nodular disease on ultrasound and/or thyroid function abnormalities, Cushing syndrome (1 mg dexamethasone suppression test was done all PCOS patients and obese control who had a BMI greater than 30), infection diseases, hypertension, hepatic or renal dysfunction were excluded from the study. The participants under the age of 18 and over the age of 35 were excluded from the study. The participants were also excluded if they had used confounding medications, including oral contraceptive agents, antilipidemic drugs, hypertensive medications, and insulin-sensitizing drugs, within 3 months before enrolment.

Weight and height were measured in light clothing without shoes. The BMI was calculated by dividing the weight by the square of the height (kg/m^2^). The waist circumference was measured at the narrowest level between the costal margin and iliac crest, and the hip circumference was measured at the widest level over the buttocks while the subjects were standing and breathing normally. The waist-to-hip ratio (WHR) was then calculated.

The degree of hirsutism was determined by the Ferriman-Gallwey (FG) score [22]. The BMI, WHR and hirsutism scores were assessed by a single investigator for all of the subjects.

### Biochemical evaluation

Venous blood samples were obtained in the follicular phase of a spontaneous or progesterone-induced menstrual cycle. Before the study, blood samples were drawn from each patient after a 12h overnight fasting for the determination of the hormones, lipid profile, high-sensitive C- reactive protein (hs-CRP), insulin and glucose levels.

Plasma glucose was determined by the glucose oxidase/peroxidase method (Gordion Diagnostic, Ankara, Turkey). The serum levels of follicle-stimulating hormone (FSH), luteinizing hormone (LH), prolactin, dehydroepiandrosterone sulphate (DHEAS), total testosterone (T), insulin, free thyroxin (fT4), thyroid stimulating hormone (TSH) and thyroid peroxidase anti-body (anti-TPO) were measured with specific electrochemiluminescence immunoassays (Elecsys 2010 Cobas, Roche Diagnostics, Mannheim, Germany). The levels of total-cholesterol, high density lipoprotein cholesterol (HDL-C), and triglyceride (TG) were determined with enzymatic colorimetric assays by spectrophotometry (BioSystems S.A., Barcelona, Spain). The low density lipoprotein cholesterol (LDL-C) level was calculated using the Friedewald formula.

Serum hs-CRP was determined using high-sensitive CRP immunonephelometry (BN, Dade-Behring; Marburg, Germany).

Insulin resistance was calculated using the homeostasis model assessment insulin resistance index (HOMA-IR) [23], according to the following formula: fasting plasma glucose (mmol/L) × fasting serum insulin (mU/mL)/22.5. The cut-off value was taken as 2.7 for HOMA-IR [24].

### Thyroid volume (TV)

Thyroid volume was assessed using a high-resolution ultrasound machine (Hitachi, Japan; EUB 7000) with a 6–14 megahertz high-frequency linear transducer. Thyroid volumes were calculated by multiplication of three diameters and the constant value 0.52.

### Statistical analyses

Collected data were entered to SPSS version 17. Continuous data were shown as means ± SD. Chi-squared tests were used to compare differences in rates. Normally distributed variables were compared using the Student T test; nonnormally distributed variables were compared by the Kolmogrow Smirnov Test. The degree of association between continuous variables was calculated by the Pearson correlation coefficient. The multiple linear regression using the stepwise method was used to determine the independent predictors.

A *p* value lower than 0.05 was accepted as statistically significant.

## Results

Clinical, biochemical and hormonal parameters were screened in the patients with PCOS and in the healthy control subjects (Table 1). We studied 47 patients with PCOS (mean age 23.27 ± 5.83 years, range 18–33 years; BMI, 24.41± 4.39 kg/m^2^) and 30 age- and BMI- matched healthy controls (mean age 22.80±4.4 years, range 18–32 years; BMI, 23.64±3.35 kg/m^2^).

**Table 1:** The clinic and biochemical data in women with polycystic ovary syndrome (PCOS) and healthy controls

	**PKOS**	**Control**	***P***
	**(n =47)**	**(n=30)**	
Age, year	23.27±5.83	22.80±4.4	0.704
BMI, kg/m^2^	24.41±4.39	23.64± 3.35	0.412
Fasting glukose, mg/dL	94.63±11.01	87.66±9.59	**0.006**
Fasting insülin, μ IU/mL	15.44± 7.98	11.34±5.52	**0.017**
HOMA-IR	2.22± 1.89	1.46±0.69	**0.034**
Total cholesterol, mg/dL	170.46±26.9	156.06±27.3	0.101
Triglyceride, mg/dL	109.29±59.46	89.13±52.82	**0.05**
HDL-C, mg/dL	54.21±12.12	53.31±14.52	0.771
LDL-C, mg/dL	95.09±23.54	84.58±24.18	0.128
hsCRP, mg/L	2.83±4.6	1.35±1.81	**0.03**

HOMA-IR: homeostasis model assessment insulin resistance index; HDL-C: high density lipoprotein cholesterol; LDL-C: Low density lipoprotein cholesterol; hs-CRP: high-sensitive C- reactive protein.

### Comparing parameters between groups

The mean fasting glucose, insulin, HOMA-IR, triglyceride, hsCRP, DHEAS, prolactin, fT4 levels were significantly higher and the estradiol levels were significantly lower in the patients with PCOS (p<0.05) (Tables 1, 2). The mean TV was 10.13±3.98 mL in PCOS patients while 10.46±4.36 mL in healthy control women (p=0.734). The number of anti-TPO positive women was similar between groups (p: 0.456).

**Table 2:** The hormonal value and tyroid volume levels in women with polycystic ovary syndrome (PCOS) and healthy controls

	**PCOS**	**Control**	**P**
	**(n = 47)**	**(n = 30)**	
fT4	1.22±0.24	1.07± 0.16	**0.007**
TSH, μ IU/ml	2.01± 1.19	1.98± 1.04	0.892
FSH, m IU/ml	5.57±2.48	6.06±2.03	0.377
LH, m IU/ml	6.63±2.69	5.12±2.66	0.121
Estradiol, pg/ml	67.71±90.85	84.48±49.16	**0.007**
Total Testosterone, ng/ml	0.48±0.21	0.41±0.11	0.09
PRL, ng/ml	20.6±13.03	12.15±5.84	**0.04**
DHEAS, μq/dl	274.3±142.3	182.23±77.6	**0.002**
Thyroid volume, ml	10.13±3.98	10.46±4.36	0.734

fT4: free thyroxine; TSH: thyroid stimulating hormone; FSH: follicule stimulating hormone; LH: luteinizing hormone; PRL: prolactin; DHEAS: dehydroepiandrosterone sulfate.

### The correlations between TV, biochemical and hormonal parameters

A significant positive correlation was found between the TV, anti-TPO and LH (r: 0.602, p<0.0001; r: 0.316, p: 0.007). However, hyperinsulinemia and insulin resistance were not found to be associated with TV. In multiple linear regression analyses TV was significantly associated with LH (beta coefficient=0.377, p<0.001), anti-TPO (beta coefficient=0.444, p<0.001) and TSH (beta coefficient= −0.309, p=0.004) (age, BMI, FSH, estradiol and HOMA-IR were included in the model).

### Comparing the TV and hormonal parameters in groups based on the HOMA-IR

The participants were divided into 2 groups according to HOMA-IR levels. The cut-off point was 2.7 [24]. Those participants who had a HOMA-IR greater than 2.7 had significantly higher hsCRP levels. Although, the mean TV measurement was higher in group with higher HOMA-IR levels, the difference was not statistically significant (Table 3).

**Table 3:** The biochemical, hormonal value and thyoid volume in HOMA-IR groups

	**HOMA-IR<2.7**	**HOMA- IR≥2.7**	***P***
fT4, ng/Dl	1.14±0.19	1.17±0.22	0.667
TSH, μ IU/ml	1.84±0.99	2.25± 1.47	0.170
hsCRP, mg/L	1.8±4.43	2.22±2.34	**0.05**
FSH, m IU/mL	5.58±2.35	6.16±2.30	0.322
LH, m IU/mL	6.09±3.93	6.44±4.99	0.735
Thyroid volume, mL	10.44±3.30	10.70±5.09	0.802

fT4: free thyroxine; TSH: thyroid stimulating hormone; hsCRP: high-sensitive C- reactive protein; FSH: follicule stimulating hormone; LH: luteinizing hormone.

**Figure 1:**
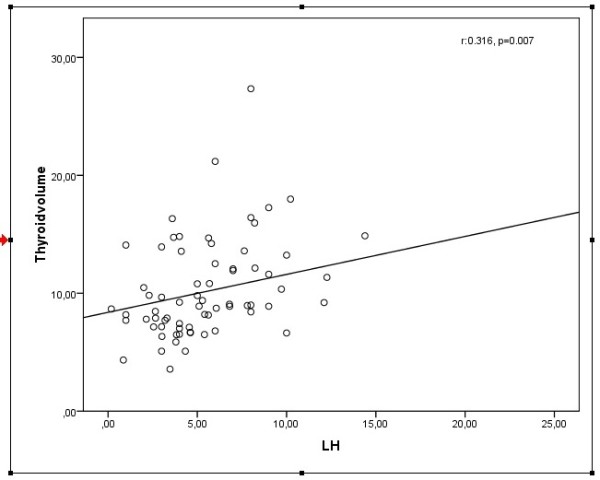
The relationship between total thyroid volume and luteinizing hormone.

The correlation between LH and TV was shown in Figure 1.

## Discussion

The results of the present study demonstrate that the mean TV measurements were similar in the early age of PCOS patients and the control group. Furthermore, insulin resistance had no effect on TV in the early age participants. However, the TV showed a significant correlation with LH and anti-TPO.

It has been shown that insulin had mitogenic effect on thyrocyte. Additionally, in recent studies hyperinsulinemia and insulin resistance has been found to be associated with TV [5,25]. However, in the present study hyperinsulinemia and insulin resistance were not found to be associated with TV. But, when we divided participants into 2 groups according to the presence of insulin resistance, we observed the higher TV measurements in insulin resistant group, but the difference was not statistically significant. It is thought to be due to lean and young participants.

Human chorionic gonadotropin (hCG) secreting placental tumours such as hydatidiform moles and choriocarcinomas have been found to be associated with hyperthyroidism [26]. Furthermore, the HCG has been reported to increase TSH receptor expression, thyroid hormone secretion, iodide uptake, organification, adenylate cyclase, and deoxyribonucleic acid synthesis in rat and also cultured human thyrocytes [27-29].

Luteinizing hormone is a glycoprotein hormone like TSH and has similar alpha subunit. In Carayon et al. study LH has been identified to increase thyroid adenylate cylase activity 65 times more potently than HCG in human thyroid membranes [12]. Also, in Yoshimura et al. study human LH has been found to be more potent than HCG in binding to the TSH receptor and stimulating adenylate cyclase [13].

As mentioned, HCG and LH have thyrotrophic effect in rat and human thyroid membrane. In present study, in consistent with these studies LH was positively correlated with TV and in regression analyses LH was obtained to be an independent risk factor for increased TV, regardless of age, BMI and anti TPO.

The higher incidence of thyroid diseases in women is previously attributed to the higher estradiol levels. Estradiol has been shown to enhance proliferative and mitogenic activities of thyroid cells [17,18]. However, recently it has been shown that chronic estradiol treatment led to significant decreases in volume densities of thyroid follicles, follicular epithelium and epithelial height [19]. In another study 3712 women were evaluated and found that oral contraceptives were related with a lower thyroid volume and reduced risk of goiter. In this study the oral contraceptives users had goitre four times more lower than non-users [20]. In this case, the LH suppression with oral contraceptives might be the factor for decreased thyroid volumes consistent with the present results. This suggests to us, endogenous elevated estradiol levels lead to increases in thyroid volume, while exogenous estradiol treatment leads to decreases in thyroid volume probably as a result of LH suppression.

The present study clearly shows that increased LH levels are associated with increased TV. Furthermore, anti-TPO, TSH and LH levels were found to be independent risk factors for increased TV. However, the lack of the association between insulin and TV might be due to lean and early age PCOS patients as well as short disease duration. Chronic stimulation with LH and insulin may lead to increase in TV in later stages of the PCOS diseases.

The study has some limitations. The sample size was small and the participants were lean and young age. The women with PCOS had higher LH levels in comparison to controls; however the difference was not significant which might be related to small sample size. Therefore, the further case control clinical studies are needed to evaluate the association between the gonadotropins and the TV.

## Competing interests

The authors declared that they have no competing interests.

## Authors' contributions

EC: have made contributions to conception and design, acquisition of data, and analysis and interpretation of data. MS: have made contributions to conception and design, acquisition of data, and analysis and interpretation of data. OT: have made contributions to acquisition of data. NC: have made contributions to acquisition of data. BK: have made contributions to acquisition of data. AG: have made contributions to acquisition of data. MA: have made contributions to acquisition of data. IU: have made contributions to acquisition of data. ET: have made contributions to acquisition of data. BU: have made contributions to acquisition of data TD: have made contributions to conception, design and interpretation of data. All authors read and approved the final manuscript.

## References

[B1] Norman RJ Dewailly D Legro RS Hickey TE Polycystic ovary syndrome Lancet 2007 370 685 697 10.1016/S0140-6736(07)61345-2 17720020

[B2] Pasquali R Gambineri A Pagotto U The impact of obesity on reproduction in women with polycystic ovary syndrome BJOG 2006 113 1148 1159 10.1111/j.1471-0528.2006.00990.x 16827825

[B3] Orio F Jr Palomba S Spinelli L Cascella T Tauchmanova L Zullo F Lombardi G Colao A The cardiovascular risk of young women with polycystic ovary syndrome: an observational, analytical, prospective case–control study J Clin Endocrinol Metab 2004 89 3696 3701 10.1210/jc.2003-032049 15292291

[B4] Kelly CJ Speirs A Gould GW Petrie JR Lyall H Connell JM Altered vascular function in young women with polycystic ovary syndrome J Clin Endocrinol Metab 2002 87 742 746 10.1210/jc.87.2.742 11836314

[B5] Yasar HY Ertugrul O Ertugrul B Ertugrul D Sahin M Insulin resistance in nodular thyroid disease Endocr Res 2011 36 167 174 10.3109/07435800.2011.593011 21973236

[B6] Kaloumenou I Alevizaki M Ladopoulos C Antoniou A Duntas LH Mastorakos G Chiotis D Mengreli C Livadas S Xekouki P Dacou-Voutetakis C Thyroid volume and echostructure in schoolchildren living in an iodine-replete area: relation to age, pubertal stage, and body mass index Thyroid 2007 17 875 881 10.1089/thy.2006.0327 17956161

[B7] Ittermann T Schmidt CO Kramer A Below H John U Thamm M Wallaschofski H Volzke H Smoking as a risk factor for thyroid volume progression and incident goiter in a region with improved iodine supply Eur J Endocrinol 2008 159 761 766 10.1530/EJE-08-0386 18765562

[B8] Cakir E Eskioglu E Aydin Y Ozkan SK Guler S Urine iodine excretion in patients with euthyroid noduler disease Ann Saudi Med 2011 31 167 170 10.4103/0256-4947.78204 21422654 PMC3102477

[B9] Boas M Hegedus L Feldt-Rasmussen U Skakkebaek NE Hilsted L Main KM Association of thyroid gland volume, serum insulin-like growth factor-I, and anthropometric variables in euthyroid prepubertal children J Clin Endocrinol Metab 2009 94 4031 4035 10.1210/jc.2009-0939 19602556

[B10] Okada M Kamiya Y Ito J Yoshimata T Kawaguchi M Shibata H Fujinami T Platelet epidermal growth factor in thyroid disorders Endocr J 1998 45 83 88 10.1507/endocrj.45.83 9625450

[B11] Pietz L Michalek K Wasko R Ruchala M Sowinski J Influence of the endogene TSH stimulation of thyroid volume increase in the patients after total thyroidectomy due to differentiated thyroid cancer Endokrynol Pol 2008 59 119 122 18465686

[B12] Carayon P Lefort G Nisula B Interaction of human chorionic gonadotropin and human luteinizing hormone with human thyroid membranes Endocrinology 1980 106 1907 1916 10.1210/endo-106-6-1907 6245856

[B13] Yoshimura M Hershman JM Pang XP Berg L Pekary AE Activation of the thyrotropin (TSH) receptor by human chorionic gonadotropin and luteinizing hormone in Chinese hamster ovary cells expressing functional human TSH receptors J Clin Endocrinol Metab 1993 77 1009 1013 10.1210/jc.77.4.1009 7691861

[B14] Arroyo A Laughlin GA Morales AJ Yen SS Inappropriate gonadotropin secretion in polycystic ovary syndrome: influence of adiposity J Clin Endocrinol Metab 1997 82 3728 3733 10.1210/jc.82.11.3728 9360532

[B15] Taylor AE McCourt B Martin KA Anderson EJ Adams JM Schoenfeld D Hall JE Determinants of abnormal gonadotropin secretion in clinically defined women with polycystic ovary syndrome J Clin Endocrinol Metab 1997 82 2248 2256 10.1210/jc.82.7.2248 9215302

[B16] Vanderpump MP Tunbridge WM French JM Appleton D Bates D Clark F Grimley Evans J Hasan DM Rodgers H Tunbridge F The incidence of thyroid disorders in the community: a twenty-year follow-up of the Whickham Survey Clin Endocrinol (Oxf) 1995 43 55 68 10.1111/j.1365-2265.1995.tb01894.x 7641412

[B17] Rajoria S Suriano R Shanmugam A Wilson YL Schantz SP Geliebter J Tiwari RK Metastatic phenotype is regulated by estrogen in thyroid cells Thyroid 2010 20 33 41 10.1089/thy.2009.0296 20067378 PMC2833180

[B18] Furlanetto TW Nguyen LQ Jameson JL Estradiol increases proliferation and down-regulates the sodium/iodide symporter gene in FRTL-5 cells Endocrinology 1999 140 5705 5711 10.1210/en.140.12.5705 10579335

[B19] Sosic-Jurjevic B Filipovic B Milosevic V Nestorovic N Manojlovic-Stojanoski M Brkic B Sekulic M Chronic estradiol exposure modulates thyroid structure and decreases T4 and T3 serum levels in middle-aged female rats Horm Res 2005 63 48 54 10.1159/000083139 15637454

[B20] Knudsen N Bulow I Laurberg P Perrild H Ovesen L Jorgensen T Low goitre prevalence among users of oral contraceptives in a population sample of 3712 women Clin Endocrinol (Oxf) 2002 57 71 76 10.1046/j.1365-2265.2002.01564.x 12100072

[B21] Rotterdam ESHRE/ASRM-Sponsored PCOS Consensus Workshop Group Revised 2004 consensus on diagnostic criteria and long-term health risks related to polycystic ovary syndrome Fertil Steril 2003 81 19 25 10.1016/j.fertnstert.2003.10.004 14711538

[B22] Ferriman D Gallwey JD Clinical assessment of body hair growth in women J Clin Endocrinol Metab 1961 21 1440 1447 10.1210/jcem-21-11-1440 13892577

[B23] Matthews DR Hosker JP Rudenski AS Naylor BA Treacher DF Turner RC Homeostasis model assessment: insulin resistance and beta-cell function from fasting plasma glucose and insulin concentrations in man Diabetologia 1985 28 412 419 10.1007/BF00280883 3899825

[B24] Gokcel A Ozsahin AK Sezgin N Karakose H Ertorer ME Akbaba M Baklaci N Sengul A Guvener N High prevalence of diabetes in Adana, a southern province of Turkey Diabetes Care 2003 26 3031 3034 10.2337/diacare.26.11.3031 14578235

[B25] Ayturk S Gursoy A Kut A Anil C Nar A Tutuncu NB Metabolic syndrome and its components are associated with increased thyroid volume and nodule prevalence in a mild-to-moderate iodine-deficient area Eur J Endocrinol 2009 161 599 605 10.1530/EJE-09-0410 19633072

[B26] Yoshimura M Hershman JM Thyrotropic action of human chorionic gonadotropin Thyroid 1995 5 425 434 10.1089/thy.1995.5.425 8563483

[B27] Hershman JM Role of human chorionic gonadotropin as a thyroid stimulator J Clin Endocrinol Metab 1992 74 258 259 10.1210/jc.74.2.258 1730804

[B28] Hershman JM Lee HY Sugawara M Mirell CJ Pang XP Yanagisawa M Pekary AE Human chorionic gonadotropin stimulates iodide uptake, adenylate cyclase, and deoxyribonucleic acid synthesis in cultured rat thyroid cells J Clin Endocrinol Metab 1988 67 74 79 10.1210/jcem-67-1-74 3379138

[B29] Kraiem Z Sadeh O Blithe DL Nisula BC Human chorionic gonadotropin stimulates thyroid hormone secretion, iodide uptake, organification, and adenosine 3',5'-monophosphate formation in cultured human thyrocytes J Clin Endocrinol Metab 1994 79 595 599 10.1210/jc.79.2.595 8045981

